# Mixing Performance of a Cross-Channel Split-and-Recombine Micro-Mixer Combined with Mixing Cell

**DOI:** 10.3390/mi11070685

**Published:** 2020-07-15

**Authors:** Makhsuda Juraeva, Dong Jin Kang

**Affiliations:** School of mechanical engineering, Yeungnam University, Gyungsan 712-749, Korea; mjuraeva@ynu.ac.kr

**Keywords:** degree of mixing, dean vortex, mixing cell, cross-channel SAR (CC-SAR)

## Abstract

A new cross-channel split-and-recombine (CC-SAR) micro-mixer was proposed, and its performance was demonstrated numerically. A numerical study was carried out over a wide range of volume flow rates from 3.1 μL/min to 826.8 μL/min. The corresponding Reynolds number ranges from 0.3 to 80. The present micro-mixer consists of four mixing units. Each mixing unit is constructed by combining one split-and-recombine (SAR) unit with a mixing cell. The mixing performance was analyzed in terms of the degree of mixing and relative mixing cost. All numerical results show that the present micro-mixer performs better than other micro-mixers based on SARs over a wide range of volume flow rate. The mixing enhancement is realized by a particular motion of vortex flow: the Dean vortex in the circular sub-channel and another vortex inside the mixing cell. The two vortex flows are generated on the different planes perpendicular to each other. They cause the two fluids to change their relative position as the fluids flow into the circular sub-channel of the SAR, eventually promoting violent mixing. High vorticity in the mixing cell elongates the flow interface between two fluids, and promotes mixing in the flow regime of molecular diffusion dominance.

## 1. Introduction

Microscale fluid mixing is an important issue in many microfluidic systems, such as micro-reactors and micro-total analysis systems (μTASs). These systems are used widely in applications, including chemical synthesis, biochemical analysis, dilution of drug solutions, and sequencing nucleic acids [1]. The mixing of two or more fluids is a common process occurring in a range of micro-mixers. The flow regime in micro-mixers corresponds to laminar flow because of the geometric size of the mixers, and molecular diffusion becomes a major mechanism of mixing. Therefore, an efficient micro-mixer is critical in the design of a microfluidic system. [2,3].

The mixing mechanism of micro-mixers is classified into passive, active, or combined techniques. Active and combined micro-mixers require an external energy source other than the energy source driving fluid flow. Examples of external energy sources include electric [3,4,5], magnetic [6,7], acoustic [8], and pulsed flow sources [9,10]. Although active micro-mixers show better mixing performance, they are less preferred because of several disadvantages, such as the difficulty of integrating them into a microfluidic system, additional equipment for the extra energy source, and complex fabrication. In contrast, passive techniques suggest a geometrical structure to agitate or generate secondary flow in micro-mixers. The disturbance is expected to cause chaotic advection in microchannel flow. Therefore, they are much easier to integrate into microfluidic systems.

We can categorize passive techniques in several groups according to mixing enhancement techniques. The first group aims to modify the channel wall geometry along which the mixing of two fluids occurs. Some examples are a grooved surface [11], a herringbone wall [12], and a twisting channel wall [13,14]. The second group of passive techniques attaches obstacles to the channel walls, such as indentations and baffles [15,16], periodic geometric features [17], and a simple block in the junction [18]. 

The third group is a design of the overall structure of a micro-mixer other than a straight microchannel: either two-dimensional (2D) or three-dimensional (3D). For example, Kashid et al. [19] examined five generic microchannel designs with a focus on the region before the fluids merge. They tested five different layouts of inlet branches. Lim et al. [20] proposed a crossing manifold micro-mixer, which has a 3D microstructure with a sequential configuration of horizontally and vertically crossing tube bundles. Other examples include various split-and-recombine (SAR) micro-mixers [21]. These designs use complex channel elements, such as multiple flow passages, 3D structures, and curved or non-straight channels. Recently, some complicated 3D micro-mixers showed high mixing performance [22]. Nevertheless, some advantages of 2D micro-mixers such as simple fabrication and integration into microfluidic systems are valuable benefits. 

The SAR is a practical mechanism of micro-mixers improving the degree of mixing (DOM). For example, Ansari et al. [23] proposed a SAR micro-mixer based on unbalanced collision, which is caused by different flow rates in the sub-channels. Li et al. [24] constructed a SAR micro-mixer by dislocating the sub-channels. Hossain [25] proposed a rhombus-based micro-mixer with three unbalanced sub-channels. On the other hand, most of the SAR micro-mixers perform poorly in the range of molecular diffusion dominance. To overcome this difficulty, some studies have attempted to combine different mixing techniques such as baffles, channel contraction, obstruction, etc. For example, Sheu et al. [26] designed a SAR with tapered curved micro-channels that allowed the introduction of Dean vortex flows by the centrifugal effects. Raza et al. [27] proposed the unbalanced SAR micro-mixer combined with baffles in curved micro-channels that improved the mixing performance of similar shaped SAR micro-mixers. Bazaz et al. [28] proposed a micro-mixer combining various mixing units such as teardrop, obstruction, nozzle & pillar, and Tesla. They demonstrated that combining planar mixing units is a good design concept to build a high-performance micro-mixer.

Researchers use an experimental and/or numerical approach to study the mixing performance of a micro-mixer, according to their preference. Numerical studies on the fluid dynamic features involved in a micro-mixer have several advantages. They allow detailed visualization of the mixing process as well as the associated flow patterns such as streamlines, vortex formation, velocity vector, etc. A quantitative evaluation of the components of a micro-mixer is also easily achieved. Therefore, many researchers rely on a numerical approach to study the mixing performance of a micro-mixer. For example, Balan et al. [29] investigated the dynamics of vortex formation in the Y- and T- micro-bifurcations. They used a commercial software, FLUENT to obtain detailed flow data in micro-channels. Raza et al. [27] studied the mixing performance of an asymmetrical split and recombine micro-mixer with baffles using CFX15.0. The numerical results were used to evaluate quantitatively the mixing performance of their micro-mixer, in comparison with others. Volpe et al. [30] used the lattice Boltzmann method (LBM) to characterize the flow properties of a microfluidic device. They showed that the numerical results describe accurately the flow dynamics of a continuous size-based sorter microfluidic device. Therefore, a numerical approach is used in this paper to analyze the mixing performance of a new passive micro-mixer.

This paper proposes a new passive micro-mixing unit combining a SAR unit with a mixing cell. The SAR unit was designed to direct flow through one sub-channel across the main channel. Therefore, the present micro-mixer was called the cross-channel SAR (CC-SAR), and the flow through single sub-channel was directed to flow in the transverse direction repeatedly. A mixing cell was placed between two consecutive SARS. Each SAR section was followed by a baffled mixing cell. The mixing performance of CC-SAR was analyzed numerically in terms of the mixing index and corresponding mixing energy cost. The 3D Navier–Stokes equations were solved using the commercial software, FLUENT 19.2. The simulations were carried out for the Reynolds numbers ranging from 0.3 to 80, corresponding to volume flow rates ranging from 3.1 μL/min to 826.8 μL/min. 

## 2. Cross-Channel Split-and-Recombine (CC-SAR) Micromixer

**Figure 1:**
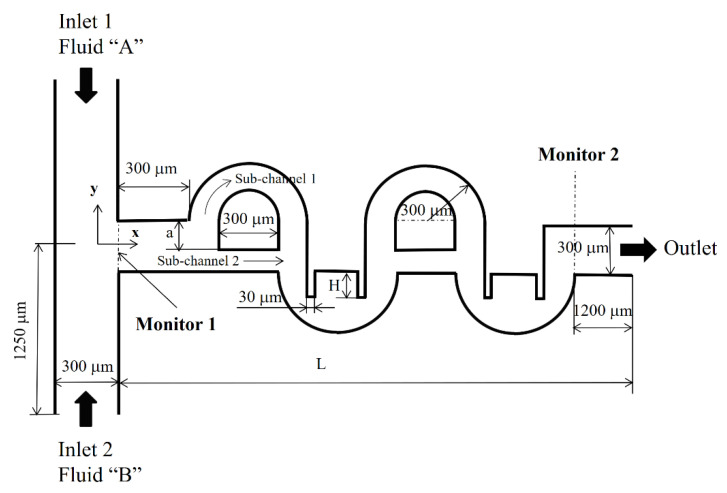
Diagram of a cross-channel split-and-recombine (CC-SAR) micro-mixer (non-proportional).

Figure 1 shows the layout of the CC-SAR micro-mixer with two mixing units. The inlets and outlet have a rectangular cross-section that is 300 μm wide and 120 μm deep. Inlets 1 and 2 are both 1250 μm long. The depth of the micro-mixer keeps constant at 120 μm. Each mixing unit consisted of one SAR with one mixing cell. The flow through sub-channel 1 was designed to flow across the main channel, while the flow along sub-channel 2 was along the main channel. The width of the sub-channel 1 is kept constant at 150 μm while that of the sub-channel 2 is varied from 60 μm to 160 μm. The geometric parameter, *a,* determines the width of the sub-channel 2 and is eventually related to the DOM in the SAR section. The width of the sub-channel 2 is (300-a) μm. As *a* is varied from 140 μm to 240 μm, the width of the sub-channel 2 varies in the range of 60 μm to 160 μm. For example, the width of the sub-channel 2 is 150 μm for *a* = 150 μm, and is equal to that of the sub-channel 1. The height of the baffles, *H*, improves the DOM in the mixing cell at the expense of the pressure load. It was varied from 150 μm to 200 μm. Two baffles are embedded in each mixing cell. The length of flow pass following the centerline of the sub-channel 1 and the mixing cell in a single mixing unit is approximately 2014 μm long. The total length of flow pass depends on the number of mixing units. In this paper, all simulations were carried out for the CC-SAR with four mixing units. If calculating along the centerline of the sub-channel 1 and the mixing cell with no baffles, the total length of flow pass from monitor 1 to monitor 2 is approximately 8055 μm. 

For the sake of simplicity, we assumed that the same aqueous solution flows into the two inlets. The properties of the fluid were assumed to be equal to those found in many existing BioMEMS systems. The density, diffusion constant and kinematic viscosity of the fluid were *ρ* = 998 kg/m^3^, *D* = 10^−10^ m^2^s^−1^ and *ν* = 10^−6^ m^2^s^−1^, respectively. This diffusion constant is a typical value for small proteins in an aqueous solution. The Schmidt (Sc) number is 10^4^ (the ratio of the kinetic viscosity and the mass diffusion of fluid). The Reynolds number is defined as $Re=\frac{\rho U_{mean}d_{h}}{\mu }$, where $\rho ,\;U_{mean},\;d_{h}\;\mathrm{and}\;\mu$ denote the density, the mean velocity at the outlet, the hydraulic diameter of main channel, and the viscosity of the fluid, respectively.

## 3. Governing Equations and Computational Procedure

The fluid was assumed to be Newtonian and incompressible, and the corresponding governing equations are the continuity and Navier–Stokes equations as follows:

(1) $$\left(\vec{u}\cdot \nabla \right)\vec{u}=-\frac{1}{\rho }\nabla p+\nu \nabla^{2}\vec{u}$$

(2) $$\nabla \cdot \vec{u}=0$$

where $\vec{u}$, *p*, and ν are the velocity vector, pressure, and kinematic viscosity, respectively. The evolution of the concentration was calculated from the advection-diffusion equation:

(3) $$\left(\vec{u}\cdot \nabla \right)\phi =D\nabla^{2}\phi$$

where *D* and *φ* are the diffusion constant and local concentration or mass fraction of a given species, respectively. 

The commercial software FLUENT 19.2. was used to solve the governing equations (Equations (1)–(3)). This code is based on the finite volume method and can model a fluid mixture. All of the convective terms in Equations (1) and (3) were approximated using the QUICK scheme (quadratic upstream interpolation for convective kinematics), which have third-order theoretical accuracy. The velocity profile at the two inlets was assumed to be uniform, while the outflow condition was specified at the outlet. For example, the velocity at the inlets is 29.3 (mm/s) for a Reynolds number of 10. The no-slip condition was specified along the other walls. The mass fraction of the fluid was set to *φ* = 1 at inlet 1 and *φ* = 0 at inlet 2.

The degree of mixing (DOM) is used to evaluate the mixing performance of CC-SAT, and is defined in the following form:

(4) $$DOM=1-\frac{1}{\xi }\sqrt{\sum_{i=1}^{n}\frac{{\left(\phi_{i}-\xi \right)}^{2}}{n}},$$

where *φ_ι_* and *n* are mass fraction in the *i^th^* cell and total number of cells, respectively. *ξ* is specified as 0.5 which indicates equal mixing of the two solutions.

The mixing energy cost (MEC) is used to evaluate the effectiveness of a micro-mixer, and is defined, combining the pressure load and DOM, as the following form [31,32]:

(5) MEC=Δpρumean2DOMX100,

where $u_{mean}$ is the average velocity at the outlet, and Δp is the pressure load between the monitor 1 and the outlet. 

## 4. Validation of the Numerical Study

**Figure 2:**
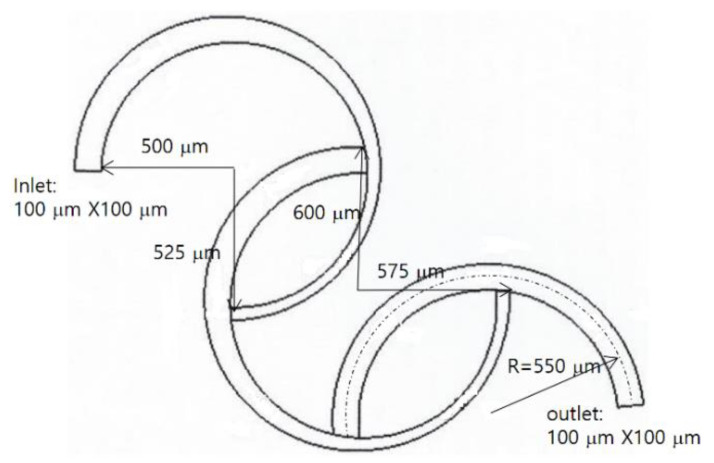
Diagram of the SAR examined by Sheu et al. [26].

The micro-mixer examined by Sheu et al. [26] was used to validate the present numerical approach. Figure 2 presents a schematic diagram of the SAR micro-mixer. It consisted of three ring-shaped channels. The second channel is connected to the first channel at 180° away from the inlet, and the third channel is connected to the second channel in the same way. The first two ring channels on the inlet side are three-quarters long, and the other ring on the outlet side is a semi-circle. The radius of curvature of the three channels is 550 μm. The cross section of the channel at the inlet and outlet is a square, 100 μm in length. The width of the first two ring channels is tapered from 100 μm to 50 μm, while its depth is kept constant at 100 μm. On the other hand, the cross section of the third channel on the outlet side remains unchanged.

Sheu et al. [26] assessed the uniformity of mixing at a specific location by defining the mixing index (MI) in the following form:

(6) $$MI=1-\frac{\sigma_{D}}{\sigma_{D,o}}$$

and

(7) $$\sigma =\sqrt{\frac{1}{n}\sum_{i=1}^{n}{\left(\phi_{i}-\phi_{ave}\right)}^{2}},$$

where $\sigma_{D}$ is the standard deviation of the concentration on a cross section normal to the flow; $\sigma_{D,o}$ is the standard deviation on the inlet cross section, and $\phi_{ave}$ is the average value of the concentration over the sampled section.

Preliminary computations were carried out to validate the present numerical approach. For this purpose, the micro-mixer experimented by Sheu et al. [26] was used in the simulation. Figure 2 presents the computational domain meshed by the structured hexahedral cells; the total number of cells ranged from 0.5 million to 1.75 million. This computation was carried out for Reynolds numbers Re = 0.5, 1, 5, and 25. 

**Figure 3:**
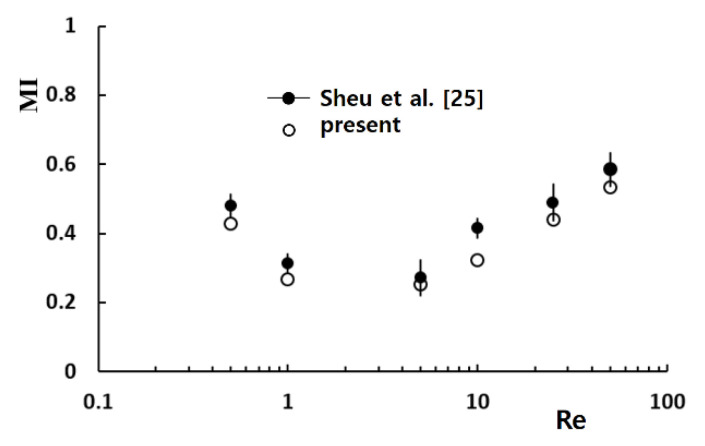
Validation of the numerical approach.

Figure 3 compares the present result with the corresponding experimental data reported by Sheu et al. [26]. The discrepancy between the numerical and experimental data is acceptable, and the variation of MI with Reynolds number is also well predicted. The discrepancy was attributed to several factors, such as numerical diffusion and experimental uncertainty.

An additional set of computations was carried out to check the grid dependence of the present simulation. This computation was made for Re = 0.5 with *a* = 190 μm and H = 200 μm. The major mixing mechanism was molecular diffusion for Re = 0.5. Therefore, the Reynolds number of 0.5 was small enough to check the effects of numerical diffusion. The computational zone was meshed by structured hexahedral cells. All computational cells had an equal size. The edge size of each cell was varied from 4 μm to 8 μm. 

**Figure 4:**
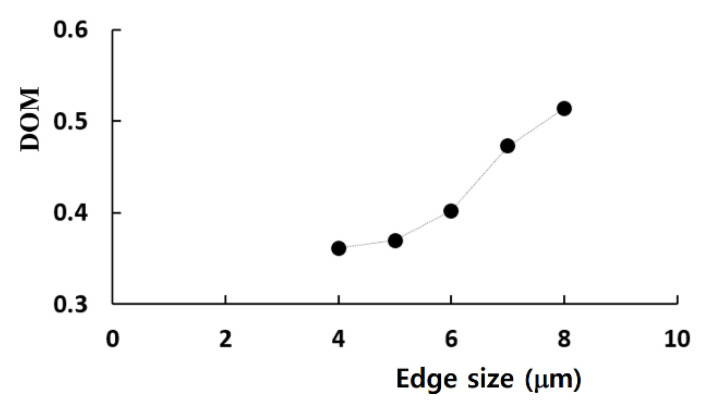
Grid dependence test.

Figure 4 shows a variation of the calculated DOM with the edge size. The DOM increased with increasing size of the computational cell, as expected. This behavior was attributed mainly to numerical diffusion. The deviation of the 5 μm and 6 μm solutions from that of the 4 μm solution was 1.4% and 9%, respectively. Therefore, 5 μm is small enough to obtain solutions with reasonable accuracy, and the total number of cells is about 2.5 million for a typical case.

The grid convergence index (GCI) was also analyzed to quantify the uncertainty of grid convergence [33]. The GCI is computed based on the Richardson extrapolation methodology defined as follows:

(8) $$GCI=F_{s}\frac{\left|\varepsilon \right|}{r^{p}-1}$$

(9) $$\varepsilon =\frac{f_{coarse}-f_{fine}}{f_{fine}},$$

where *F_s_, r*, and *p* are the safety factor of the method, grid refinement ratio, and the order of accuracy of the numerical method, respectively. *f_coarse_* and *f_fine_* are the numerical results obtained with a coarse grid and fine grid, respectively. *F_s_* was specified as 1.25 according to the suggestion by Roache [34]. We analyzed the three numerical solutions obtained with the edge size of 4 μm, 5 μm, and 6 μm. The corresponding number of nodes are 4.76 × 10^6^, 2.54 × 10^6^, and 1.24 × 10^6^, respectively. The computed GCI were reduced from 2.4% to 0.94%. Therefore, the edge size of 5 μm is confirmed as small enough to obtain numerical solutions with reasonable accuracy.

## 5. Results and Discussion

A new passive micro-mixer combining the SAR and a baffled mixing cell was proposed, and simulated to examine its mixing performance for Reynolds numbers ranging from 0.3 to 80. The velocity at the two inlets was uniform in the range from 0.88 mm/s to 234 mm/s, and the corresponding volume flow rates ranged from 3.1 μL/min to 826.8 μL/min. The degree of mixing was evaluated at the outlet, and the corresponding mixing energy cost was also assessed.

**Figure 5:**
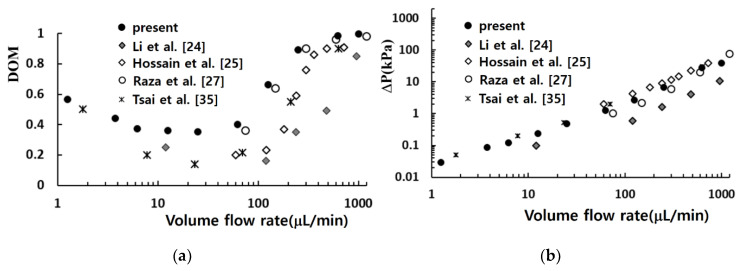
Variation of the degree of mixing (DOM) (**a**) and pressure load (**b**) with the volume flow rate.

Figure 5 compares the simulated mixing performance in terms of the DOM and the required pressure load with those from other micro-mixers. Li et al. [24] proposed a planar asymmetric SAR mixer with dislocated sub-channels, and investigated experimentally and numerically the effect of geometric parameters and the Reynolds number on mixing performance. They showed that multi-directional vortices and the Dean vortex enhanced the mixing performance of their micro-mixer. Raza et al. [27] proposed a SAR mixing unit combined with baffles in a curved channel, and analyzed numerically its mixing performance. They showed that its mixing performance is better than their earlier version of SAR micro-mixer. Hossain et al. [25] designed a micro-mixer with unbalanced three-split rhombic sub-channels, and used a numerical approach to analyze its mixing performance. The results show that a rhombic angle of 90° gave the best mixing performance. Tsai et al. [35] studied experimentally and numerically the mixing performance of a micro-mixer based on baffles in a curved channel. According to their results, the multi-directional vortices and the converging-diverging flow caused by baffles contribute together to the enhancement of mixing.

**Figure 6:**
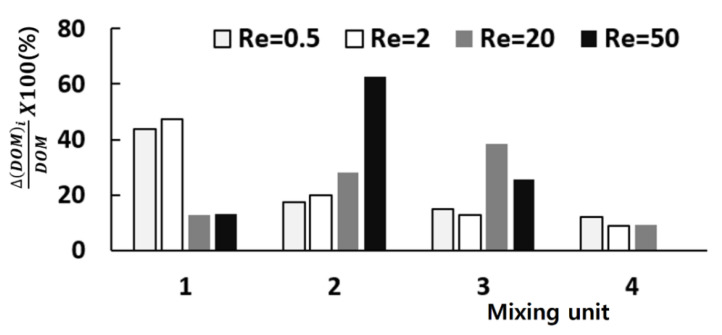
DOM obtained in each mixing unit.

Figure 6 shows how each mixing unit performs in terms of DOM for the Reynolds numbers Re = 0.5, 2, 20, and 50. It compares how much of mixing is obtained in each mixing unit. The vertical ordinate represents the DOM increment in each mixing unit (Δ(DOM)_i_ in the plot), and its value means a percentage of DOM with respect to the total amount of DOM. For low Reynolds number flows such as Re = 0.5 and 2, the first and second mixing units perform better than the remaining units. However, the mixing happens more vigorously in the second and third mixing units for high Reynolds number flows such as Re = 20 and 50. One thing to note is that the fourth mixing unit contributes nothing for the Reynolds number of 50. This suggests that the number of mixing units can be optimized in terms of DOM.

**Figure 7:**
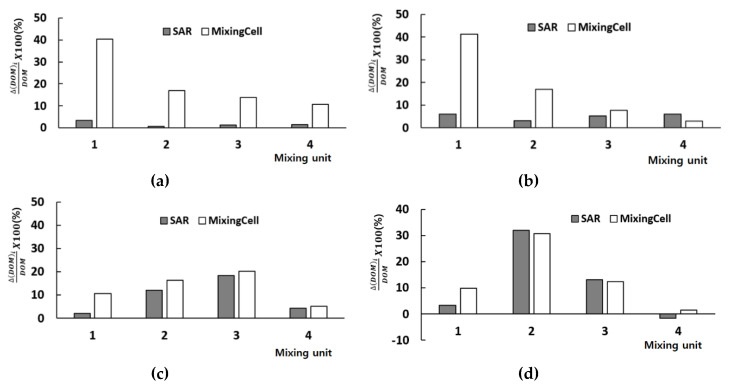
DOM obtained in SAR and mixing cell of each mixing unit: Re = 0.5 (**a**) Re = 2 (**b**) Re = 20 (**c**) Re = 50 (**d**).

As each mixing unit is comprised of one SAR and one mixing cell, their role is also evaluated. Figure 7 shows how each SAR and mixing cell performs for the Reynolds numbers Re = 0.5, 2, 20 and 50. Here, Δ(DOM)_i_ indicates the DOM increment in either the SAR or the mixing cell. Figure 7 confirms that the mixing cell is an excellent mixing device over a wide range of the Reynolds number. For the Reynolds numbers Re = 0.5, 2, and 20, in particular, the mixing cell contributes to mixing more than the SAR. This explains why the present micro-mixer performs better than other SAR based micro-mixers as shown in Figure 5. The SAR becomes dominant for the Reynolds number larger than about 50.

A set of simulations was carried out for the Reynolds numbers of 1, 10, 20, 50, and 80 to determine how an unequal flow rate in the sub-channels of the micro-mixer affects the mixing performance. The control parameter *a* was varied in the range, 140 μm to 220 μm, to control the volume flow rate, whereas the height of the baffle was kept at 150 μm. 

**Figure 8:**
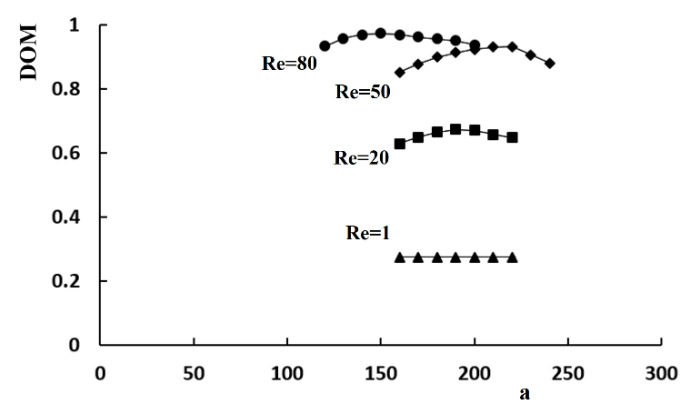
Variation of the DOM with the control parameter, *a*.

Figure 8 shows the variation of the DOM with respect to the control parameter *a*. The effects of an unequal flow rate on the mixing performance were noticeable for the Reynolds numbers larger than about 20. This behavior coincides with the finding reported in other SAR micro-mixers. The unbalanced collision at the recombine section promoted mixing [23,25]. Therefore, the control parameter *a* is a reasonable design parameter to control the flow rate in a split section and can be optimized in terms of the DOM. On the other hand, the optimal value of *a* is dependent on the Reynolds number. Moreover, the effects of an unequal flow rate on the mixing performance are negligible for Re = 1, where the molecular diffusion is dominant. This suggests that a secondary mechanism is necessary to improve the mixing performance in the low flow rate regime.

**Figure 9:**
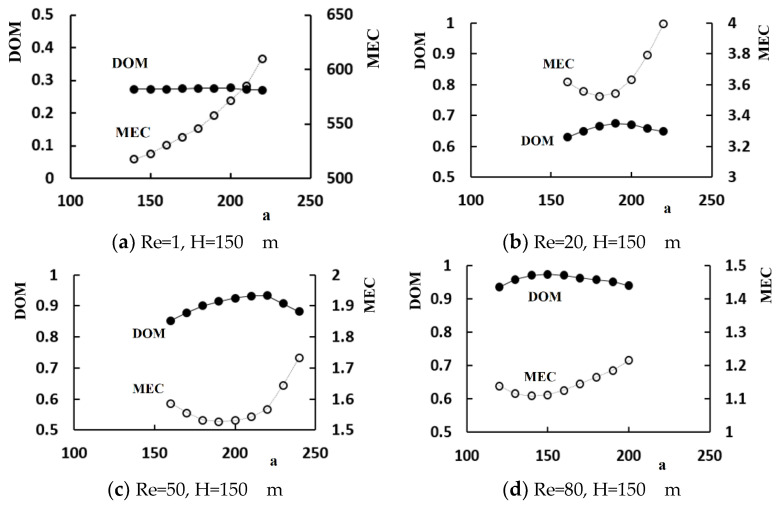
Variation of the DOM and MEC with the control parameter, *a*.

Figure 9 compares the variation of MEC with the DOM in terms of the design parameter *a*. The MEC is also optimized as a function of the design parameter *a*. For example, the optimal ratio of the mass flow rate through the straight sub-channel (sub-channel 2 in Figure 1) to the entire flow rate is approximately 74% and 71% for Re = 20 and 50, respectively. On the other hand, the optimal value of *a* is different from that for the DOM. An increase in *a* leads to a monotonic increase in the MEC without any improvement of the DOM for Re = 1. This confirms that the unequal flow rate in the sub-channels of a SAR unit is not an effective mechanism to enhance mixing in the molecular diffusion regime.

**Figure 10:**
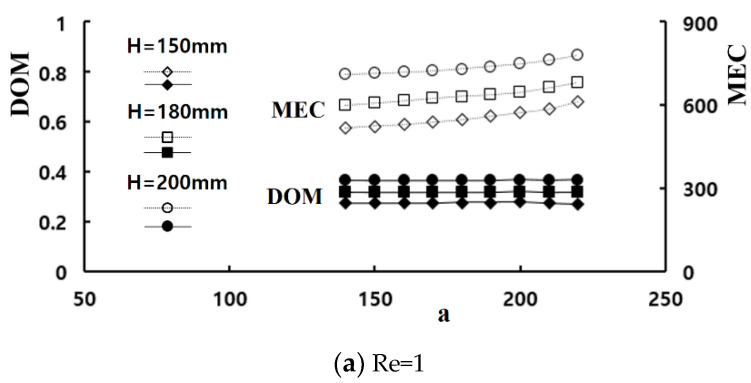

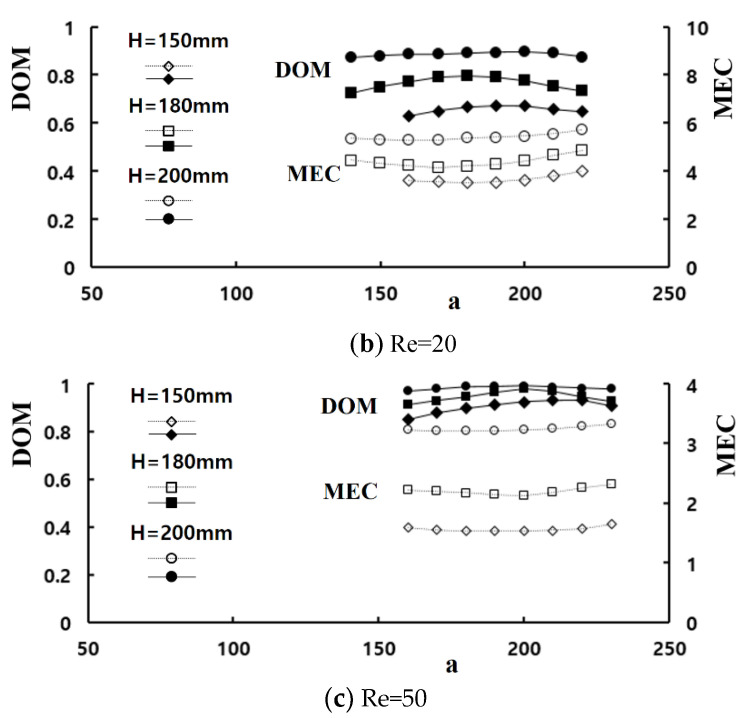
Variation of the DOM and MEX with the control parameter *a*.

A set of computations were carried out by varying the height of the baffle embedded in the mixing cells to examine how the baffle improves the mixing for Re = 1, 20, and 50. Figure 10 presents the results of DOM improvement with the required pressure load. Comparing Figure 10a with Figure 10b,c, an increment of the baffle height causes much larger increment of MEC as the Reynolds number increases. In other words, a larger value of H results in mixing enhancement, but also requires much larger pressure load as the Reynolds number increases. For example, when the baffle height is increased from 150 μm to 200 μm, the improvements of the DOM are approximately 32% and 33% for Re = 1 and 20, respectively. Meanwhile, the corresponding increment of pressure load are approximately 0.1 kPa and 3.4 kPa, and are 77% and 104% increments, respectively. Therefore, Re = 20 requires much larger percentage increment of pressure load for a similar percentage increment of DOM. For Re = 50, the improvement of DOM is only 5.1% as the baffle height is increased from 150 μm to 200 μm. However, the percentage increment of pressure load is 43%. Therefore, the height of baffle should be determined considering required pressure load. For example, we can obtain the DOM of 0.93 for Re = 50 with either H = 150 μm or 180 μm. However, the corresponding pressure loads are 12.3 kPa and 17.5 kPa, respectively.

**Figure 11:**
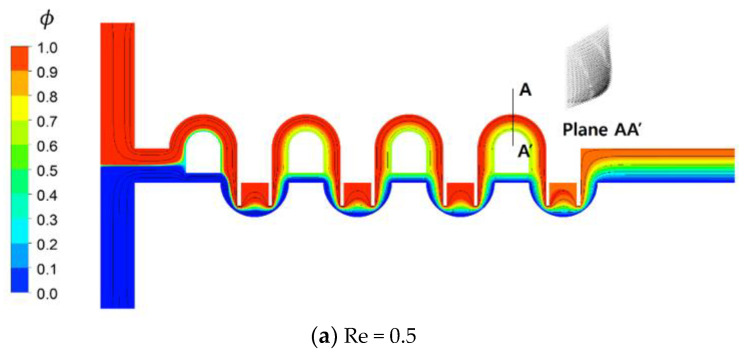

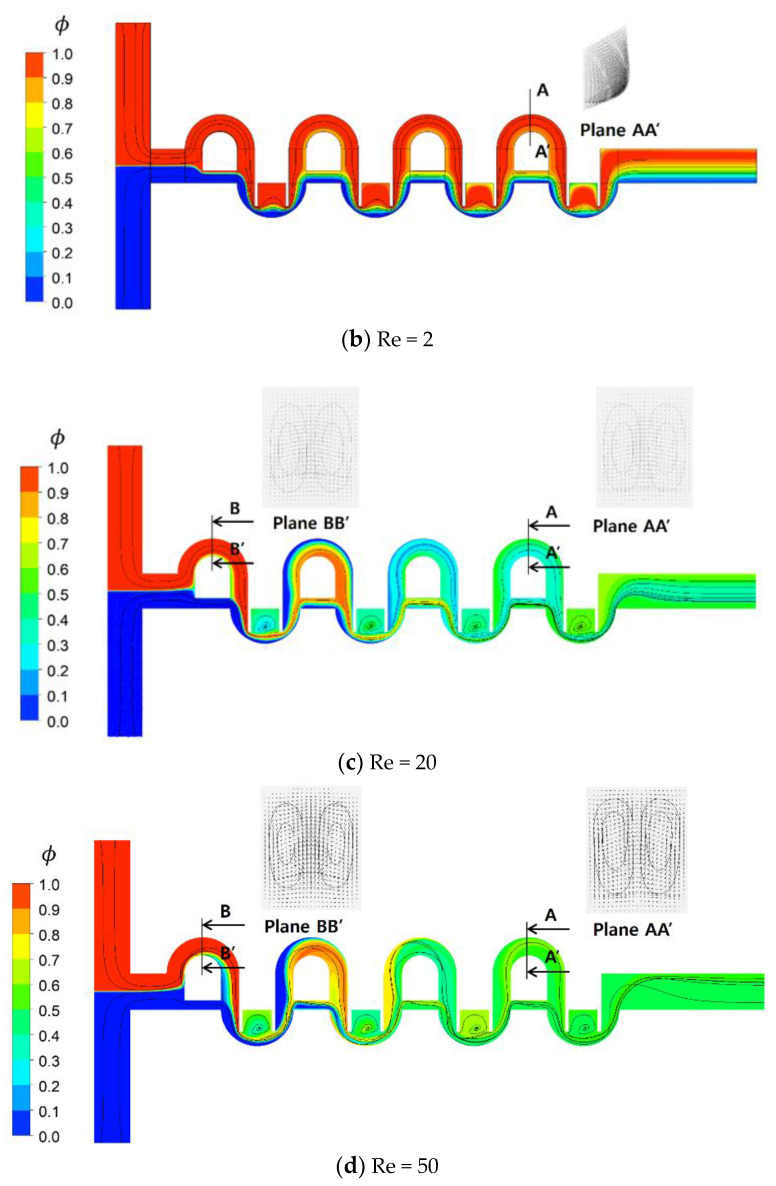
Projected streamlines and contours of the concentration distribution on the plane of the channel half.

Figure 11 shows the distribution of mass fraction of the fluid A on the plane of channel half width z = 60 μm for the Reynolds numbers Re = 0.5, 2, 20, and 50. The two counter-rotating vortices in Figure 11c,d are the Dean vortex flow, and are due to the centrifugal force along sub-channel 1. Another vortex flow forms inside the mixing cells for Re = 20 and 50. This vortex flow is in the z-direction, perpendicular to that of the Dean vortex. This indicates that the vortex flow changes its direction as it moves from the mixing cell into the circular sub-channel of the SAR unit. Figure 11c,d shows that fluids A (red in the figure) and B (blue in the figure) in the mixing cell change their position as they flow into the circular sub-channel of the SAR unit. This explains why the present micro-mixer shows excellent mixing performance over a wide flow range of mixing regimes.

**Figure 12:**
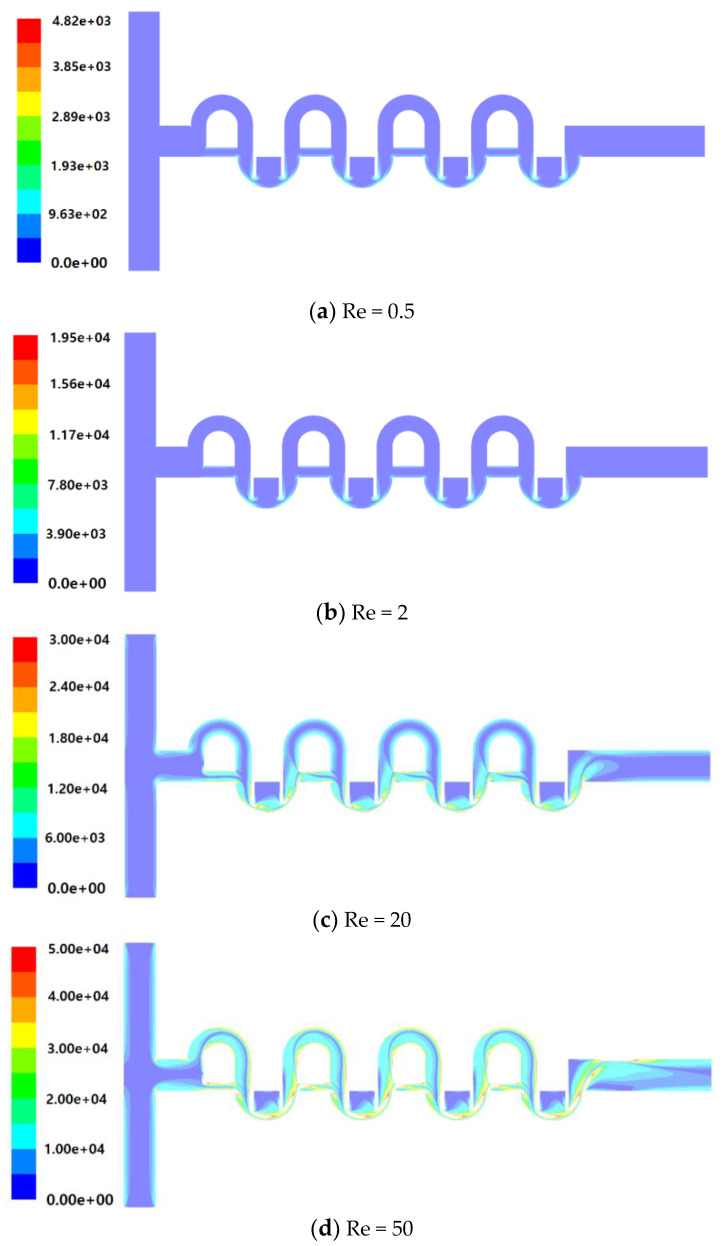
Distribution of vorticity magnitude on the plane of the channel half width z = 60 μm.

Figure 12 shows the distribution of vorticity magnitude on the plane of channel half width z = 60 μm for the Reynolds numbers Re = 0.5, 2, 20, and 50. The vorticity is intensive around the baffles in the mixing cell and/or along the two sub-channels of the SAR. For low Reynolds numbers such as Re = 0.5 and 2, the vorticity in the sub-channels of the SAR is so weak that there is no meaningful DOM increment in the SAR as seen in the Figure 7a,b. However, the intensive vorticity in the mixing cell causes the streamlines to follow closely the baffle walls, as can be seen in the Figure 11a,b. By contrast, the vorticity magnitude in the sub-channels of the SAR is comparable with that in the mixing cell for high Reynolds numbers such as Re = 20 and 50. This results in the Dean vortex shown in Figure 11c,d.

## 6. Conclusions

A new passive micro-mixer was designed combining SARs with mixing cells, and two baffles were embedded in each mixing cell. Each SAR has two sub-channels: circular and straight. The flow through the circular sub-channel is designed to cross the main channel, and meets the flow through the straight sub-channel at a right angle. The mixing performance of the present micro-mixer was evaluated numerically by examining the DOM and MEC. The numerical simulation was carried out for Reynolds numbers ranging from 0.3 to 80.

A comparison with other micro-mixers based on SARs shows that the present micro-mixer performs better over the whole range of Reynolds numbers. Therefore, the mixing enhancement is obtained in the flow regime of molecular diffusion dominance as well as the convection-dominant flow regime. By contrast, the corresponding pressure load is similar to those required by other micro-mixers.

The mixing enhancement is attributed to a particular motion of vortex flow: two vortex flows form and change their direction. One vortex flow is formed inside the mixing cell, and its direction of rotation is perpendicular to the main stream. The other one is the Dean vortex formed in the circular sub-channel of the SAR, and its direction of rotation is in the main stream. This direction change causes the two fluids A and B to change their relative position as they flow into the circular sub-channel and eventually promotes mixing violently. High vorticity in the mixing cell elongates the flow interface between two fluids, and eventually promotes mixing in the flow regime of molecular diffusion dominance.

The mass flow rate through the circular sub-channels can be optimized in terms of the MEC and DOM. The optimal value of *a* depends on the Reynolds number. The height of the baffle in the mixing cell promotes mixing at the expense of the pressure load. For example, as the baffle height is increased from 150 μm to 200 μm, the improvement of the DOM are approximately 32%, 33%, and 5.1% for Re = 1, 20 and 50, respectively. Meanwhile, the corresponding increments of pressure load are also approximately 0.1 kPa, 3.3 kPa and 5.4 kPa, respectively. Even if the baffle height is a good design parameter to enhance the DOM, the height of the baffle should be determined considering required pressure load and the Reynolds number.

The mixing cell performs better than the SAR for low Reynolds numbers less than about 50, and their mixing performance is reversed for high Reynolds numbers larger than about 50. Therefore, the combination of a SAR with a mixing cell is a practical design concept to devise a micro-mixer working over a wide range of the Reynolds number. 

The present cross-channel SAR is simple and performs better than other SAR micro-mixers over a wide range of the volume flow rates. It can be integrated into a variety of applications, such as cell cultures, microfluidic filtration, and biochemistry analysis. 

## Nomenclature

*a*	width of split block of SAR (μm)
*D*	diffusion coefficient (m^2^/s)
*DOM*	degree of mixing
*f_coarse_*	numerical solution obtained with coarse grid
*f_fine_*	numerical solution obtained with fine grid
*GCI*	grid convergence index
*H*	height of baffle (μm)
*n*	total number of sampling points at a specific plane
*MI*	mixing index
*MEC*	mixing energy cost
Δp	pressure load (Pa)
*r*	grid refinement ratio
*Re*	Reynolds number
*SAR*	split and recombine
*U_mean_*	average velocity at the outlet (m/s)
*x, y, z*	Cartesian coordinates
**Greek symbols**	
*ε*	relative error
*μ*	fluid viscosity (kg/(ms))
*ν*	fluid kinematic viscosity (m^2^/s)
*φ*	mass fraction
*ρ*	fluid density (kg/m^3^)
σ	standard deviation
**Subscripts**	
*i*	sampling point
